# The Valuable Reference of Live Birth Rate in the Single Vitrified-Warmed BB/BC/CB Blastocyst Transfer: The Cleavage-Stage Embryo Quality and Embryo Development Speed

**DOI:** 10.3389/fphys.2020.01102

**Published:** 2020-09-10

**Authors:** Xi Shen, Hui Long, Hongyuan Gao, Wenya Guo, Yating Xie, Di Chen, Yanyan Cong, Yun Wang, Dongying Li, Jiqiang Si, Leiwen Zhao, Qifeng Lyu, Yanping Kuang, Li Wang

**Affiliations:** Department of Assisted Reproduction, Shanghai Ninth People’s Hospital Affiliated to Shanghai Jiao Tong University School of Medicine, Shanghai, China

**Keywords:** frozen embryo transfer, grade “C” blastocyst, cleavage-stage embryo quality, embryo development speed, live birth rate

## Abstract

**Background:**

It is unclear whether we should focus attention on cleavage-stage embryo quality and embryo development speed when transferring single particular grade vitrified-warmed blastocysts, especially poor-quality blastocysts (grade “C”).

**Method:**

This retrospective study considered 3386 single vitrified-warmed blastocyst transfer cycles from January 2010 to December 2017. They were divided into group 1 (AA/AB/BA, *n* = 374), group 2 (BB, *n* = 1789), group 3 (BC, *n* = 901), and group 4 (CB, *n* = 322). The effects of cleavage-stage embryo quality and embryo development speed were measured in terms of clinical pregnancy and live birth rates in each group.

**Results:**

Pregnancy outcomes showed a worsening trend from groups 1 to 4; the proportion of embryos with better cleavage-stage quality and faster development speed decreased. In group 1, only the blastocyst expansion degree 3 was a negative factor in the clinical pregnancy rate (odds ratio (OR) [95% confidence interval (CI)]: 0.233 [0.091–0.595]) and live birth rate (0.280 [0.093–0.884]). In the other groups (BB, BC, and CB), blastocysts frozen on day 5 had significantly better clinical pregnancy outcomes than those frozen on day 6: 1.373 [1.095–1.722] for group 2, 1.523 [1.055–2.197] for group 3, and 3.627 [1.715–7.671] for group 4. The live birth rate was 1.342 [1.060–1.700] for group 2, 1.544 [1.058–2.253] in group 3, and 3.202 [1.509–6.795] in group 4, all *P*s < 0.05). The degree of blastocoel expansion three for clinical pregnancy rate in group 2 (0.350 [0.135–0.906], *P* < 0.05) and day 3 blastomere number (>7) for live birth rate in group 4 (2.455 [1.190–5.063], *P* < 0.05) were two important factors.

**Conclusion:**

We should consider choosing BB/BC/CB grade blastocysts frozen on day 5, CB grade blastocysts with day 3 blastomere numbers (>7), and AA/AB/BA grade blastocysts with degrees of expansion (≥4) to obtain better pregnancy outcomes.

## Introduction

Blastocyst transfer in *in vitro fertilization* (IVF) could yield higher implantation and pregnancy rates than cleavage-stage embryo transfer (Gardner et al., 1998a,b; Schwarzler et al., 2004). Furthermore, blastocyst-stage transfer involves the selection of more highly developed embryos. This ultimately reduces multiple birth rates because fewer embryos are required to achieve a pregnancy. These facts suggest that it is important to choose the blastocysts with the highest developmental potential. Various kinds of blastocyst grading schemes have emerged and evolved in clinical practice (Cohen et al., 1985; Rehman et al., 2007; Bodri et al., 2015; Richardson et al., 2015; Wirleitner et al., 2016); nevertheless, the majority of them today are based on the Gardner system (Gardner and Schoolcraft, 1999). The parameters in the Gardner system encompass expansion grades (scores 1–6), individual evaluation of the inner cell mass (ICM) as well as trophectoderm (TE; grades A, B, and C). This remains the method most often implemented globally. High blastocyst quality has been proven to be associated with higher probability of success, in either fresh or vitrified-warmed embryo transfer (Rehman et al., 2007; Guerif et al., 2009, 2010; Roy et al., 2014).

When blastocysts reach the same grade, it is unclear whether we should be focusing on cleavage-stage embryo quality and embryo development speed before transfer. The success rates of good-quality blastocyst (≥3 BB) transfer appear uncompromised by embryos of different quality at the cleavage stage in a good-prognosis population (Herbemont et al., 2017). However, as far as we know, no study has previously assessed the effects of the detailed cleavage-stage morphology and embryo development speed on single vitrified-warmed blastocyst transfer outcomes, especially in low-quality blastocysts (stages 1 or 2, or ≥3 with a grade “C” ICM or TE) (Morbeck, 2017).

Grade “C” blastocysts are usually transferred in fresh cycles, but their utilization in frozen embryo transfer is limited. Grade “C” blastocysts are usually euploid (Capalbo et al., 2014; Christodoulou et al., 2017) and can generate live births with normal obstetric and perinatal outcomes (Wirleitner et al., 2016; Bouillon et al., 2017). With the increasing number of freeze-all cycles, the judgment accuracy of borderline blastocysts becomes increasingly important. The decision of whether to freeze these borderline blastocysts remains subjective among embryologists. A recent study illustrated that the inter-rater agreement is fair when accessing poor-quality embryos (Hammond et al., 2020) and leads us to explore additional parameters for freezing borderline blastocysts. Therefore, in the present study, we explore the reference values of cleavage-stage embryo quality and embryo development speed in terms of live birth rates in single vitrified-warmed blastocyst transfer, not solely in grade “C” blastocysts, but also in AA/AB/BA, BB grade blastocysts.

## Materials and Methods

### Study Design and Participants

This study was exempted from institutional review board approval because of its retrospective nature and the use of an anonymous database. No identifiable data were available to the researchers. The current retrospective study was performed between January 2010 and December 2017 at the Department of Assisted Reproduction of the Ninth People’s Hospital affiliated with Shanghai Jiao Tong University School of Medicine. Women undergoing frozen embryo transfer (FET) cycles with single blastocyst transfer were included. Exclusion criteria were as follows: vanishing twin syndrome or congenital uterine malformations. Patients who underwent day 7 blastocyst transfers or blastocyst blastocoel B1 and B2 transfers were excluded, the numbers of which are fewer and may lead to bias.

### Laboratory Protocols

The controlled ovarian hyperstimulation (COH) regimen was previously described (Wang et al., 2016, 2018). All aspirated oocytes were transferred to modified human tubal fluid (HTF) medium (Irvine Scientific, United States) and then transferred to culture medium. According to semen parameters, insemination was carried out via either conventional insemination (IVF) or intracytoplasmic sperm injection (ICSI) (Henkel and Schill, 2003).

It is worth mentioning that, before 2013, embryos were cultured in early cleavage medium (Irvine Scientific, United States) before day 3 and then transferred in multiblast medium (Irvine Scientific, United States). Since 2013, all embryos have been cultured in continuous single culture (Irvine Scientific, United States) (Du et al., 2017). Cleavage-stage embryo quality was evaluated on day 3 according to the Cummins’ standard (Cummins et al., 1986). Embryo morphology was scored as grade I, no cytoplasmic fragmentation; grade II, less than 20% fragmentation; grade III, 20–50% fragmentation; grade IV, greater than 50% fragmentation. The number of cells per cleavage-stage embryo was also recorded (Cummins et al., 1986). The embryologists who perform cleavage-stage embryo and blastocyst grading are experienced, resulting in lower inter-embryologist and intra-embryologist variations to the greatest extent possible for top and non-viable/degenerate quality blastocysts (Storr et al., 2017; Martinez-Granados et al., 2018; Hammond et al., 2020). The good-quality cleavage-stage embryos were defined as at least 8-cell grade II embryos. In our center, if the patients have fewer than six good-quality cleavage embryos, all good-quality cleavage embryos are frozen. Otherwise, if they have more than six, the redundant good-quality embryos are used for blastocyst culture. Embryos with poor quality were placed in extended culture until they reached the blastocyst stage and then frozen.

Blastocyst morphology was evaluated according to Gardner and Schoolcraft’s classification (Gardner and Schoolcraft, 1999), according to blastocoel, inner cell mass, and TE. The blastocysts in this study were classified into four groups: group 1 (AA/AB/BA), group 2 (BB), group 3 (BC), and group 4 (CB). The vitrification procedure was conducted by the Cryotop carrier system (Kitazato Biopharma Co.) and the cryoprotectant solution consisted of 15% (v/v) ethylene glycol, 15% (v/v) dimethyl sulfoxide, and 0.5 M sucrose. When thawing blastocysts, sequential 1, 0.5, and 0 M sucrose solutions were used as the cryoprotectant dilutions. All these steps were carried out at room temperature except for the first warming step (37°C) (Kuang et al., 2015). The check time point for cleavage-stage embryos and blastocysts was between 8 and 10 am.

### Embryo Transfer and Luteal Support

According to the individual condition of the patient, a modified natural cycle or artificial cycle was used in endometrium preparation (Zhang et al., 2019). Summarily, a modified natural cycle was suggested for patients with menstrual regularity, human chorionic gonadotropin (hCG, 5000 IU; Lizhu Pharmaceutical Trading Co., Shanghai, China) was used as a trigger. Luteal progestin supplement included Femoston tablets (4 mg/day, Abbott Healthcare Products B.V.) and soft vaginal progesterone capsules (0.4 g/day, Utrogestan, Laboratories Besins Iscovesco, France).

Artificial cycles were recommended in patients with menstrual irregularity or abnormal vaginal bleeding history. Oral 17β-estradiol (Fematone 2 mg, three times daily) was commenced from the third day onward for 14 days, and luteal support was described as above.

### Statistical Analysis

The primary outcome was the live birth rate. The secondary outcome was clinical pregnancy rate. The following definitions were used: Live births were defined as at least 22 gestational weeks or at least 500 g. Live birth rate was defined as a live birth per embryo transfer cycle. Clinical pregnancy rate: (intrauterine + ectopic pregnancies)/the number of transfer cycles. Miscarriage rate was calculated as numbers of miscarriage per clinical pregnancy cycle (Zegers-Hochschild et al., 2017).

Statistical analyses were carried out using SPSS software (SPSS, version 22, SPSS Inc., Chicago, IL, United States) and R software (version 3.6.1). Variables were expressed as the means ± SD in tables, tested using the Student *t*-test or one-way ANOVA. Qualitative data were expressed as percentages and were tested using the chi-squared test or Fisher’s exact test when appropriate. To control for repeated observations of the same patients, if the same patient fell into different groups, these patients were excluded. If the repeated cycles in one patient were in one group, the generalized estimated equations (GEE) model was applied to quantify the relevant factors of embryonic development in terms of clinical pregnancy rate and live birth rate in the four blastocyst groups. The adjusted odds ratio (aOR) was calculated using the significant confounding factors (*P* < 0.15) selected from age, infertility duration, body mass index (BMI), infertility types (primary or secondary), infertility reasons (female, male, combined, or unknown reasons), number of 2PN, number of frozen blastocysts, insemination method (IVF or ICSI), endometrial thickness, endometrial preparation (modified natural cycles or hormone therapy cycles) and treatment year (2010–2012, 2013–2014, or 2015–2017). We also noted previous FET times (0–1 or ≥2) as an important confounding factor because some patients had blastocyst transfer after cleavage-stage embryo transfer. A *P* value < 0.05 was referred to as a statistical significance.

## Results

### Four Blastocyst Grade Groups Had Different Clinical Pregnancy Outcomes and Composition Proportions of Embryos With Different Cleavage-Stage Quality and Development Speed

**FIGURE 1:**
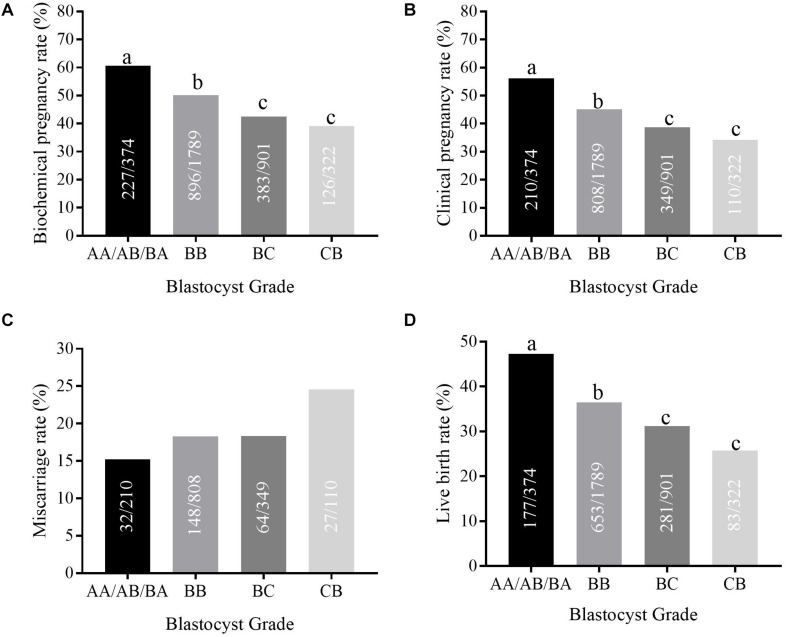
The clinical pregnancy outcomes in different blastocyst grade groups. **(A–D)** shows the biochemical pregnancy rate, clinical pregnancy rate, implantation rate, and live birth rate in different blastocyst grade groups (AA/AB/BA, BB, BC, and CB group). Different letters represent significant difference (*P* < 0.05).

The highest biochemical pregnancy rate was observed in group 1 (AA/AB/BA, 60.70%), compared with group 2 (BB, 50.08%), group 3 (BC, 42.51%), and group 4 (CB, 39.13%) (Figure 1). The same trend was found for the clinical pregnancy rate (56.15% vs. 45.16% vs. 38.73% vs. 34.16%, *P* < 0.05) and the live birth rate (47.33% vs. 36.50% vs. 31.19% vs. 25.78% *P* < 0.05). *Post hoc* analysis shows that these rates in the AA/AB/BA group were higher than in the BB group; rates in the BB group were higher than those of the BC and CB groups. The miscarriage rate increased from group 1 to group 4 (15.24% vs. 18.32% vs. 18.34% vs. 24.55%); however, this was not statistically significant.

Table 1 displays the composition proportions in the four blastocyst grade groups in relation to cleavage-stage embryo quality and embryo development speed. The proportion of day 3 blastomere cell number <6 increased (25.94% vs. 33.93% vs. 41.73% vs. 36.64%, respectively, *P* < 0.05) from group 1 (AA/AB/BA) to group 2 (BB) to group 3 (BC) to group 4 (CB); however, the proportion of day 3 blastomere cell number ≥ 8 (26.20% vs. 20.51% vs. 15.54% vs. 20.19%, respectively, *P* < 0.05) and the grade I–II percentage (24.60% vs. 12.58% vs. 8.32% vs. 7.76%, respectively, *P* < 0.05) decreased. The embryo development speed showed a slower trend across these four groups, which is the proportion of blastocysts frozen on day 6 increased (48.93% vs. 71.83% vs. 83.46% vs. 88.51%, respectively, *P* < 0.05). *Post hoc* analysis shows that the AA/AB/BA group had the best parameters, and the BC or CB group had the worst. A significant difference, as denoted by different superscript letters in Table 1, can be seen. The degrees of expansion differed among these groups (*P* < 0.05). The other essential parameters of patients in groups 1–4 are described in Supplementary Table S1.

**TABLE 1:** The constitution proportions of embryos with different cleavage-stage quality and development speed in four blastocyst grade groups.

	Group 1 AA/AB/BA (*n* = 374)	Group 2 BB (*n* = 1789)	Group 3 BC (*n* = 901)	Group 4 CB (*n* = 322)	*P*
***Day 3 assessment (cell number), n (%)***					
<6	97(25.94)^a^	607(33.93)^b^	376(41.73)^c^	118(36.64)^bc^	<0.001
6–7	179(47.86)	815(45.56)	385(42.73)	139(43.17)	0.291
≥8	98(26.20)^a^	367(20.51)^a^	140(15.54)^b^	65(20.19)^ab^	<0.001
***Day 3 assessment (embryo grade), n (%)***					
I–II	92(24.60)^a^	225(12.58)^b^	75(8.32)^c^	25(7.76)^bc^	<0.001
III–IV	282(75.40)^a^	1564(87.42)^b^	826(91.68)^c^	297(92.24)^bc^	<0.001
***Blastocyst frozen day, n (%)***					
Day 5	191(51.07)^a^	504(28.17)^b^	149(16.54)^c^	37(11.49)^c^	<0.001
Day 6	183(48.93)^a^	1285(71.83)^b^	752(83.46)^c^	285(88.51)^c^	<0.001
***The degree of expansion, n (%)***					
3	27(7.22)^a^	31(1.73)^b^	30(3.33)^b^	6(1.86)^a^	<0.001
4	310(82.89)^a^	1591(88.94)^b^	835(92.67)^c^	277(86.03)^ab^	<0.001
5	25(6.68)^a^	119(6.65)^a^	30(3.33)^b^	25(7.76)^a^	0.002
6	12(3.21)^a^	48(2.68)^a^	6(0.67)^b^	14(4.35)^a^	<0.001

*Different letters represent significant difference *(P* < 0.05).*

### The Reference Value of Cleavage-Stage Embryo Quality and Embryo Development Speed for Live Birth Rate in AA/AB/BA, BB, BC, and CB Grade Blastocysts

Table 2 displays the reference value of cleavage-stage embryo quality and embryo development speed for the live birth rate in four blastocyst grade groups, including day 3 embryo cell number, day 3 embryo grade, blastocyst frozen day (days 5 or 6), and the degree of expansion (3, 4, 5, or 6). The confounding factors of live birth rate used in Table 2 are derived from the analysis in Supplementary Table S2. Among the best AA/AB/BA grade blastocysts (group 1), only the blastocyst expansion degree 3 (vs. 4) showed negative crude OR (0.221 [0.082–0.600]) and adjusted OR (0.280 [0.093–0.884]). However, the BB grade blastocysts in group 2 (1.342 [1.060–1.700]) and BC grade blastocysts in group 3 (1.544 [1.058–2.253]) with blastocyst frozen on day 5 resulted in higher aOR of live birth rate than did day 6. For CB grade blastocysts in group 4, the aOR of live birth rate was significantly higher in blastocysts frozen on days 5 than 6 (3.202 [1.509–6.795]) and day 3 embryo cell number with more than 7 (2.455 [1.190–5.036], *P* < 0.05).

**TABLE 2:** Crude and adjusted OR of live birth rate in different grade blastocysts.

		Crude OR (95% CI)	*P*	Adjusted OR (95% CI)	*P*
		
		Group 1 (AA/AB/BA)			
Day 3 assessment (cell number)	6–7 (vs. < 6)	1.161(0.708–1.906)	0.554	1.222(0.713–2.095)	0.466
	More than 7 (vs. < 6)	1.206(0.686–2.118)	0.515	1.356(0.706–2.606)	0.361
Day 3 assessment (embryo grade)	I–II (vs. III–IV)	0.724(0.448–1.170)	0.187	0.782(0.453–1.350)	0.378
Blastocyst frozen day	Day 5 (vs. Day 6)	0.983(0.653–1.480)	0.936	0.901(0.553–1.466)	0.674
Degree of expansion	3 (vs. 4)	0.221(0.082–0.600)	0.003	0.280(0.093–0.884)	**0.024**
	5 (vs. 4)	0.766(0.336–1.744)	0.525	0.825(0.356–1.909)	0.653
	6 (vs. 4)	0.487(0.144–1.652)	0.248	0.533(0.165–1.722)	0.293

		**Group 2 (BB)**			

Day 3 assessment (cell number)	6–7 (vs. < 6)	1.282(1.028–1.598)	0.027	1.165(0.923–1.471)	0.198
	More than 7 (vs. < 6)	1.226(0.936–1.606)	0.138	1.083(0.804–1.460)	0.598
Day 3 assessment (embryo grade)	I–II (vs. III–IV)	0.873(0.649–1.173)	0.366	0.856(0.618–1.187)	0.352
Blastocyst frozen day	Day 5 (vs. Day 6)	1.406(1.138–1.736)	0.002	1.342(1.060–1.700)	**0.014**
Degree of expansion	3 (vs. 4)	0.254(0.088–0.730)	0.011	0.352(0.114–1.089)	0.070
	5 (vs. 4)	1.120(0.767–1.635)	0.559	1.153(0.776–1.715)	0.481
	6 (vs. 4)	0.858(0.467–1.576)	0.620	0.966(0.519–1.796)	0.913

		**Group 3 (BC)**			

Day 3 assessment (cell number)	6–7 (vs. < 6)	1.134(0.833–1.544)	0.424	1.053(0.766–1.447)	0.749
	More than 7 (vs. < 6)	1.168(0.771–1.771)	0.464	1.132(0.737–1.736)	0.571
Day 3 assessment (embryo grade)	I–II (vs. III–IV)	0.787(0.462–1.342)	0.379	0.855(0.481–1.519)	0.593
Blastocyst frozen day	Day 5 (vs. Day 6)	1.565(1.088–2.252)	0.016	1.544(1.058–2.253)	**0.024**
Degree of expansion	3 (vs. 4)	0.804(0.353–1.830)	0.604	0.933(0.376–2.317)	0.881
	5 (vs. 4)	1.106(0.511–2.394)	0.799	1.169(0.540–2.532)	0.691
	6 (vs. 4)	2.212(0.443–11.031)	0.333	2.393(0.567–10.096)	0.235

		**Group 4 (CB)**			

Day 3 assessment (cell number)	6–7 (vs. < 6)	1.124(0.617–2.049)	0.702	1.136(0.594–2.174)	0.700
	More than 7 (vs. < 6)	2.964(1.525–5.762)	0.001	2.455(1.190–5.063)	**0.015**
Day 3 assessment (embryo grade)	I–II (vs. III–IV)	0.902(0.348–2.342)	0.833	0.781(0.190–3.206)	0.732
Blastocyst frozen day	Day 5 (vs. Day 6)	3.206(1.589–6.468)	0.001	3.202(1.509–6.795)	**0.002**
Degree of expansion^a^	5 (vs. 4)	0.495(0.165–1.487)	0.210	0.525(0.162–1.706)	0.284
	6 (vs. 4)	0.433(0.095–1.979)	0.280	0.439(0.094–2.062)	0.297

*a: *P* value could not be calculated in expansion degree 3 subgroup because there are only six patients without live births (live birth rate = 0/6). Crude OR = 3.751E-13, Adjusted OR = 7.948E-14. The bold P values of adjusted OR means statistical significance.*

We analyze the reference value of cleavage-stage embryo quality and embryo development speed for clinical pregnancy rate in four blastocyst grade groups in Supplementary Table S3. The confounding variables of clinical pregnancy rate used in Supplementary Table S3 derive from the analysis in Supplementary Table S4. The degree of blastocoel expansion (degree 3) was a negative parameter for clinical pregnancy rate (0.350 [0.135–0.906]) for the BB group, which was different from the live birth rate.

### The Clinical Pregnancy Rate and Live Birth Rate of Selected BB/BC/CB Blastocysts Based on Positive Reference Value of Cleavage-Stage Embryo Quality and Embryo Development Speed Significantly Surpassed Its Original Level

Table 3 summarizes reference values of cleavage-stage embryo quality and embryo development speed for clinical pregnancy rate and live birth rate in different grade blastocysts. For AA/AB/BA blastocysts, the embryonic factors, including day 3 embryo cell number, day 3 embryo grade, and blastocyst frozen day (days 5 or 6), had no impact on either the clinical pregnancy rate or the live birth rate, but the degree of expansion (degree 3) gave rise to lower rates. However, for BB, BC, and CB blastocysts, those frozen on day 5 had significantly better clinical pregnancy rates and live birth rates than those frozen on day 6. Except for those frozen on day 5, the day 3 blastomere number (>7) for the CB group in live birth rate and the blastocyst degree (≥4) for the BB group in clinical pregnancy rate were also important factors.

**TABLE 3:** The summarized reference values of cleavage-stage embryo quality and embryo development speed for clinical pregnancy rate and live birth rate in different grade blastocysts.

	Group 1 AA/AB/BA (*n* = 374)		Group 2 BB (*n* = 1789)		Group 3 BC (*n* = 901)		Group 4 CB (*n* = 322)	
				
	Clinical pregnancy rate	Live birth rate	Clinical pregnancy rate	Live birth rate	Clinical pregnancy rate	Live birth rate	Clinical pregnancy rate	Live birth rate
***Day 3 assessment (cell number)***								
<6	Ref	Ref	Ref	Ref	Ref	Ref	Ref	Ref
6–7	–	–	–	–	–	–	–	–
≥8	–	–	–	–	–	–	–	↑
***Day 3 assessment (embryo grade)***								
I–II	–	–	–	–	–	-	-	–
III–IV	Ref	Ref	Ref	Ref	Ref	Ref	Ref	Ref
***Blastocyst frozen day***								
Day 5	–	–	↑	↑	↑	↑	↑	↑
Day 6	Ref	Ref	Ref	Ref	Ref	Ref	Ref	Ref
***Degree of expansion***								
3	↓	↓	↓	–	–	–	a	a
4	Ref	Ref	Ref	Ref	Ref	Ref	Ref	Ref
5	–	–	–	–	–	–	–	–
6	–	–	–	–	–	–	–	–

*a means the odds ratio is too small and P value could not be calculated due to the clinical pregnancy rate(0/6) and live birth rate (0/6) in this subgroup; ↑ represents for positive predictor; ↓ represents for negative predictor.*

**FIGURE 2:**
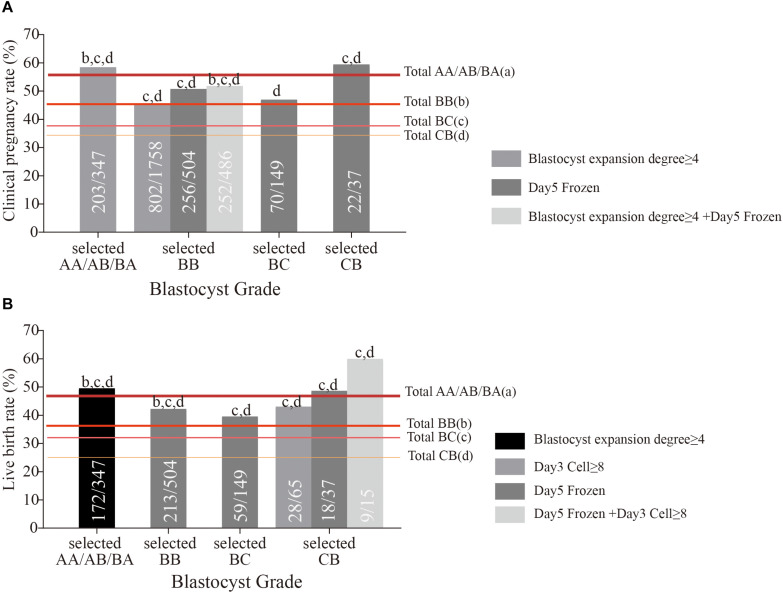
The clinical pregnancy rate **(A)** and live birth rate **(B)** of selected AA/AB/BA, BB, BC, and CB grade blastocysts based on positive reference of cleavage-stage embryo quality and embryo development. The letters above the column represent that the clinical pregnancy outcomes of selected grade blastocysts had a significant difference (*P* < 0.05) with total AA/AB/BA (a), BB (b), BC (c), and CB (d) grade blastocysts.

We selected the eligible blastocysts in each grade according to the positive reference values of cleavage-stage embryo quality and embryo development speed (Table 3) for measurement of clinical pregnancy and live birth rates. For example, we selected the eligible BB blastocysts (frozen on day 5) from all BB grade blastocysts. Surprisingly, as seen in Figure 2, these selected BB grade blastocysts achieved a similar clinical pregnancy rate (50.79%) and live birth rate (42.26%) to AA/AB/BA grade blastocysts, far beyond the original level. The selected BC blastocysts with blastocysts frozen on day 5 also achieved a similar clinical pregnancy rate (46.98%) and live birth rate (39.60%) as AA/AB/BA and BB grade blastocysts. The most surprising finding was that selected CB blastocysts frozen on days 5 and 3 embryo cell number (more than 7) reached the live birth rate of AA/AB/BA grade blastocysts (Figure 2B). The age of patients was not significantly different between the total sample and group selected according to the positive reference values of cleavage-stage embryo quality and embryo development speed (Supplementary Figure S1A). The age of patients with and without live birth was also compared; no significant difference was found in all composite groups and their separate selected groups (Supplementary Figures S1B–E). This indicates that using these positive references could select the eligible BC/CB grade blastocysts within all different age groups.

## Discussion

We found that clinical pregnancy outcomes worsened from AA/AB/BA, BB, BC to CB grade blastocysts, and the proportion of embryos with better cleavage-stage embryo quality and faster development speed decreased. Among the best AA/AB/BA grade blastocysts, neither the number of blastomeres, nor the degree of cytoplasmic fragmentation on day 3 had any effect on the pregnancy outcomes. Only the blastocysts expansion stage 3 was a negative factor. Among BB/BC grade blastocysts, higher clinical pregnancy rates and live birth rates were observed if we chose the blastocysts frozen on day 5. Furthermore, the selected CB grade blastocysts having day 3 embryo cell number with more than 7 and blastocysts frozen on day 5 had higher clinical pregnancy rates.

### Strength and Limitation

The total number of single blastocyst frozen-thaw transfer (FET) cycles in this study was 3386, broken down as group 1 (AA/AB/BA, *n* = 374), group 2 (BB, *n* = 1789), group 3 (BC, *n* = 901), and group 4 (CB, *n* = 322). This study had the largest sample size to date, especially for the BC/CB grade blastocysts. We were the first to determine whether and how we should refer to cleavage-stage embryo quality and embryo development speed in single vitrified-warmed AA/AB/BA, BB, BC, or CB grade blastocyst transfer so as to select blastocysts with better developmental potential and higher clinical pregnancy and live birth rates.

To the best of our knowledge, no previous study has focused on whether and how the individual cleavage-stage embryo quality and embryo developmental speed should be referenced for grade “C” blastocysts. We were the first to find that BC grade blastocysts having blastocyst frozen on day 5 and CB grade blastocysts having blastocyst frozen on day 5 and cleavage-stage embryo cell numbers with more than 7 achieved the similar live birth rates as AA/AB/BA grade blastocysts. This is of great importance for patients only achieving poor-quality blastocysts. It will also help us identify the relative implantation potential of blastocysts with borderline quality.

There are also some limitations. First, the retrospective nature and relatively small sample size in AA/AB/BA and CB grade blastocysts may lead to some biases. In group 1, we put AA, AB, and BA into a single group because the number of each grade of blastocysts was small, and this may have led to a reduction of statistical power. This is due to the high-quality cleavage-stage embryos (at least 8-cell, grade II) that were cultured to blastocyst only when patients in our center had more than six high-quality cleavage embryos. The blastocysts in our study did not come from a full cohort, which may cause some biases. Further evaluation of the day 3 prediction value should be conducted using a full blastocyst culture program. Second, we only evaluated single blastocyst transfers although we do also perform double-blastocyst transfer. We did not evaluate these transfers because there are too many combinations of varying blastocyst quality, thereby resulting in fewer patients in each group. Last, we only focused on vitrified-warmed transfer in our study. We mainly used the COH protocol (progestin-primed ovarian stimulation), which is combined with FET (Massin, 2017), resulting in a limited number of fresh blastocyst transfers in our center. Although there was blastocyst morphology parameter that exhibited a significant ability to predict live birth in both fresh and vitrified-warmed single blastocyst transfer cycles, that is, the degree of blastocoele expansion and reexpansion (Du et al., 2016), it is difficult to predict if there is going to be a trend similar to that which we describe in fresh single blastocyst transfers without further research.

### Different Results of Ours From Other Research

Previous studies focus on the diversity of clinical pregnancy outcomes after transplantation of varying grades of blastocyst. It is well established that blastocyst grade positively correlates with clinical outcomes (Rehman et al., 2007). Nevertheless, until now, regarding transferring a particular grade of blastocyst, it has been unclear as to whether and how we should refer to the cleavage-stage embryo quality and embryo development speed. Few studies have focused on this point. Especially for grade “C” blastocysts with borderline quality, there has been scarcely any exploration of its implantation potential.

In our study, parameters in cleavage-stage morphology (blastomere number and day 3 grade) had no reference value for cleavage-stage embryo quality on pregnancy outcomes in groups 1 and 2. This is consistent with the results of a previous study (Herbemont et al., 2017). That study concludes that good-quality blastocyst transfer should be carried out irrespective of embryo quality at cleavage stage in a good-prognosis population. The authors enrolled patients with transferred blastocyst grade ≥ 3 BB; by contrast, in the present study, we further classify blastocyst grade AA/AB/BA and BB. Furthermore, their study classifies cleavage-stage embryos as good- and poor-quality embryos roughly, whereas in our study, several factors are used to comprehensively evaluate the reference value of cleavage-stage embryo quality, including the number of blastomeres and degree of cytoplasmic fragmentation on day 3. Except for cleavage-stage embryo quality, we add embryo development speed as another reference and find that the blastocyst expansion degree 3 (compared with 4) is a negative factor in pregnancy outcomes of AA/AB/BA grade blastocysts.

Embryo development speed significantly predicts outcomes for BB/BC/CB grade blastocysts: there are higher clinical pregnancy and live birth rates in blastocysts that developed to BB/BC/CB grade and were frozen on day 5 (D5) than those on day 6 (D6). Several studies focus on D5 or D6 blastocyst transfer and conclude that D5 blastocyst transfer is better than D6 (Shapiro et al., 2001; Barrenetxea et al., 2005). However, their population was mainly concentrated in fresh embryo transfer, in which there may be desynchrony between embryo and endometrium for those D6 blastocysts developing slowly. To reduce this confounding factor, the FET method permits transfer of these slowly developing D6 blastocysts on day 5 and finds that pregnancy outcomes of D5 blastocysts are still better than D6 blastocysts. In their study, the detailed proportions of various grade blastocysts are not provided (Richter et al., 2016) and the significant differences of various grade blastocysts are noted between D5 and D6 groups (Du et al., 2018); therefore, the better pregnancy outcomes may be attributed to embryonic development speed or to the higher proportion of better grade blastocysts in the D5 blastocyst group. A meta-analysis demonstrates higher pregnancy outcomes of D5 blastocysts (better than 3 BB) than D6; however, no significant differences were found when they were at the same development stage (Sunkara et al., 2010). Therefore, in our study, we place emphasis on the same grade blastocyst group and analyze the reference value of the embryo development rate. We further verify that D5 blastocysts have better pregnancy outcomes than D6 blastocysts in BB, BC, and CB grade blastocysts. This may provide more clarity for clinical application because BB grade blastocysts are common.

#### Mechanisms of Positive Reference Values Within Cleavage-Stage Embryo Quality and Embryo Developing Speed for Clinical Pregnancy Outcomes

For BB, BC, and CB grade blastocysts, our study also shows that the blastocysts frozen on day 5 had better clinical pregnancy outcomes than those frozen on day 6. The possible explanation may be that growth retardation in blastocysts is due to genetic abnormalities. However, this hypothesis is controversial. Some studies indicate that slower-growing blastocysts have higher incidences of aneuploidy (Hashimoto et al., 2013; Taylor et al., 2014), and others find that faster and slower growing blastocysts have similar aneuploidy rates (Hashimoto et al., 2013). There are scarce studies focusing on D5 and D6 embryos in poor-quality blastocysts; we believe that, in poor-quality embryos, growth retardation may result in higher abnormal rates than in good-quality embryos. Further validation can be confirmed using preimplantation genetic diagnosis.

The number of cleavage-stage blastomeres has a positive reference value for live birth rate in CB grade blastocysts. It was previously thought that those with fewer than five cells would be discarded. However, extended embryo culture techniques have been widely used, giving these embryos more development potential. Some studies find that patients with fewer blastocysts in day 3 embryos have lower blastocyst formation rates (Ertzeid et al., 2003); however, once blastocysts are obtained (≥3 BB), they have the same developmental potential as others (Zhao et al., 2015). This is similar to our findings in the clinical pregnancy rate of groups 1–4; however, for group 4 (CB blastocyst), the patients with more than seven cleavage blastomeres were more likely to obtain a live birth. Fewer cleavage-stage blastomeres may be associated with a higher possibility of abnormal chromosomes (Hardarson et al., 2003).

Blastocoel expansion degree is also an important factor for the pregnancy outcomes in groups 1 and 2 (AA/AB/BA/BB). Some studies of blastocoel expansion degree have suggested that, in fresh or frozen transplantation, the degree of blastocoel expansion is related to the clinical pregnancy outcome, and the blastocyst with the degree of expansion grade 4 has the best outcomes in vitrified-warmed cycles (Du et al., 2016); this is similar to our results. However, there were very few patients (6 patients) and no live births with blastocyst expansion degree 3 in the CB group, so this factor needs further investigation in the future.

## Conclusion

Cleavage-stage embryo quality and embryo development speed significantly influence clinical pregnancy outcomes in single frozen BB, BC, and CB grade blastocyst transfers. Blastocysts frozen on day 5 had positive effects on single frozen BB, BC, and CB blastocyst transfer. Blastocoel expansion grade ≥4 for the AA group and cleavage-stage blastomere number more than 7 on day 3 for the CB group also had positive effects.

## Data Availability Statement

The datasets generated for this study are available on request to the corresponding author.

## Ethics Statement

Ethical review and approval was not required for the study on human participants in accordance with the local legislation and institutional requirements. The patients/participants provided their written informed consent to participate in this study.

## Author Contributions

LW, YK, and QL supervised the entire study, including the procedures, conception, design, and completion. XS and HL conducted the acquisition of data, analysis, interpretation of data, and drafting the manuscript. WG, YX, DC, HG, YC, YW, DL, JS, and LZ conducted acquisition of data and revised the article. All authors approved the final version of the manuscript.

## Conflict of Interest

The authors declare that the research was conducted in the absence of any commercial or financial relationships that could be construed as a potential conflict of interest.

## Supplementary Material

FIGURE S1 The comparison of age between total and their selected group **(A)**. The age between patients with and without live births were compared in the BB group **(B)**, BC group **(C)**, and CB group **(D)**. Total patients (black), selected patients with blastocyst expansion degree ≥4 (magenta), selected patients with day 5 frozen (red), selected patients with day 3 cell ≥8 (blue), and selected patients with day 5 frozen combined with day 3 cell ≥8 (green) are shown as boxplots in panels **(A–E)**. The difference between total patients and selected patients is compared using Student’s *t*-test or one-way ANOVA.
