# Supplementary figures and images for: The Valuable Reference of Live Birth Rate in the Single Vitrified-Warmed BB/BC/CB Blastocyst Transfer: The Cleavage-Stage Embryo Quality and Embryo Development Speed
Source: Front Physiol. 2020 Sep 10;11:1102. doi: 10.3389/fphys.2020.01102 (PMC7511572; doi:10.3389/fphys.2020.01102)

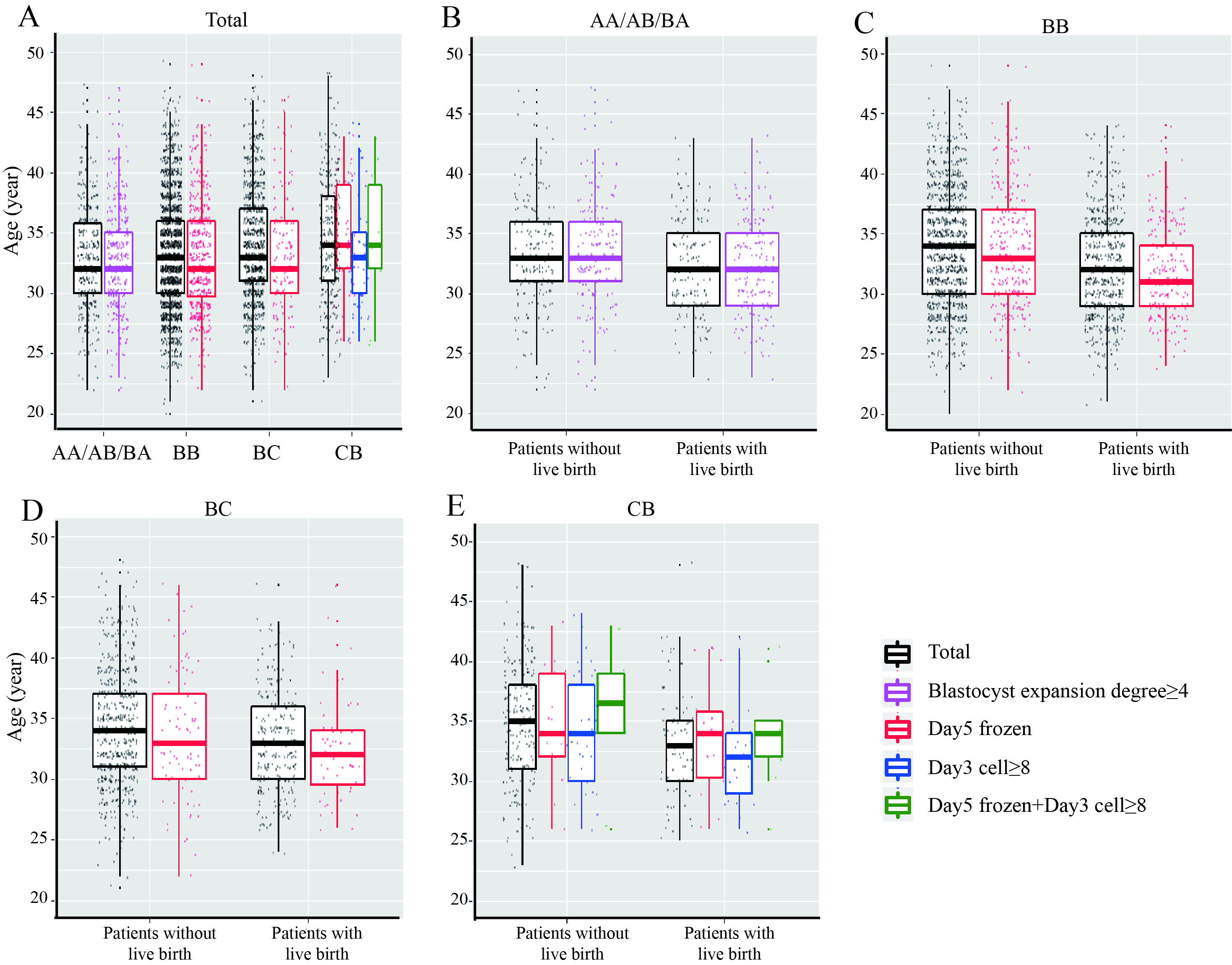

Supplement: FIGURE S1 — The comparison of age between total and their selected group (A). The age between patients with and without live births were compared in the BB group (B), BC group (C), and CB group (D). Total patients (black), selected patients with blastocyst expansion degree ≥4 (magenta), selected patients with day 5 frozen (red), selected patients with day 3 cell ≥8 (blue), and selected patients with day 5 frozen combined with day 3 cell ≥8 (green) are shown as boxplots in panels (A–E). The difference between total patients and selected patients is compared using Student’s t-test or one-way ANOVA. [file Image_1.jpg]
