# Supplementary material for: The Valuable Reference of Live Birth Rate in the Single Vitrified-Warmed BB/BC/CB Blastocyst Transfer: The Cleavage-Stage Embryo Quality and Embryo Development Speed
Source: Front Physiol. 2020 Sep 10;11:1102. doi: 10.3389/fphys.2020.01102 (PMC7511572; doi:10.3389/fphys.2020.01102)
Supplement: Supplementary file 2 [file Table_1.docx]

| Supplement Table 1. The clinic parameters of patients in all groups | | | | | |
| --- | --- | --- | --- | --- | --- |
|  | Group1  AA/AB/BA  (*n*=374) | Group2  BB  (*n*=1789) | Group3  BC  (*n*=901) | Group4  CB  (*n*=322) | *P* |
| Age(year) | 32.82±4.26 ^a^ | 33.26±4.63 ^a^ | 33.80±4.64 ^b^ | 34.46±4.86 ^b^ | <0.001 |
| Duration of infertility (years) | 3.36±2.69 ^a^ | 3.65±3.17 ^ab^ | 3.53±3.09 ^ab^ | 4.04±3.77 ^b^ | 0.028 |
| BMI (kg/m^2^) | 21.24±2.86 ^a^ | 21.79±3.01^b^ | 21.47±3.05 ^ab^ | 21.74±2.91^ab^ | 0.022 |
| Infertility type |  |  |  |  | 0.702 |
| Primary infertility | 52.94(198/374) | 50.59(905/1789) | 50.72(457/901) | 48.45(156/322) |  |
| Secondary infertility | 47.06(176/374) | 49.41(884/1789) | 49.28(444/901) | 51.55(166/322) |  |
| Infertility reason |  |  |  |  | 0.557 |
| Female | 64.71(242/374) | 61.04(1092/1789) | 61.38(553/901) | 56.83(183/322) |  |
| Male | 9.89(37/374) | 11.96(214/1789) | 10.77(97/901) | 13.35(43/322) |  |
| Combined | 10.16(38/374) | 10.29(184/1789) | 10.54(95/901) | 9.32(30/322) |  |
| Unknown | 15.24(57/374) | 16.71(299/1789) | 17.31(156/901) | 20.50(66/322) |  |
| Number of 2PN (n) | 6.90±5.15 ^a^ | 5.97±4.24 ^b^ | 5.23±3.78 ^c^ | 4.85±3.42 ^c^ | <0.001 |
| Number of frozen blastocysts (n) | 1.50±1.27 ^a^ | 1.29±0.86 ^b^ | 1.13±0.53 ^c^ | 1.09±0.44 ^c^ | <0.001 |
| Insemination method |  |  |  |  | 0.076 |
| IVF | 74.33(278/374) | 70.21(1256/1789) | 67.15(605/901) | 68.94(222/322) |  |
| ICSI | 25.67(96/374) | 29.79(533/1789) | 32.85(296/901) | 31.06(100/322) |  |
| Endometrial thickness (mm) | 10.34±2.25 | 10.33±2.30 | 10.33±2.22 | 10.30±2.16 | 0.995 |
| Endometrial preparation |  |  |  |  | 0.009 |
| Modified natural cycles | 71.66(268/374) ^a^ | 65.01(1163/1789) ^ab^ | 61.71(556/901) ^b^ | 63.98(206/322) ^ab^ |  |
| Hormone therapy cycles | 28.34(106/374) | 34.99(626/1789) | 38.29(345/901) | 36.02(116/322) |  |
| FET times |  |  |  |  | 0.741 |
| 0-1 | 77.27(289/374) | 77.08(1379/1789) | 78.58(708/901) | 79.19(255/322) |  |
| ≥2 | 22.73(85/374) | 22.92(410/1789) | 21.42(193/901) | 20.81(67/322) |  |
| Treatment of year |  |  |  |  | <0.001 |
| 2010-2012 | 20.06(75/374) ^a^ | 8.72(156/1789) ^b^ | 9.88(89/901) ^b^ | 5.59(18/322) ^b^ |  |
| 2013-2014 | 37.43(140/374) | 37.90(678/1789) | 26.64(240/901) | 26.40(85/322) |  |
| 2015-2017 | 42.51(159/374) | 53.38(955/1789) | 63.48(572/901) | 68.01(219/322) |  |

Note：Different alphabet means significant difference between groups
