# Supplementary material for: The Valuable Reference of Live Birth Rate in the Single Vitrified-Warmed BB/BC/CB Blastocyst Transfer: The Cleavage-Stage Embryo Quality and Embryo Development Speed
Source: Front Physiol. 2020 Sep 10;11:1102. doi: 10.3389/fphys.2020.01102 (PMC7511572; doi:10.3389/fphys.2020.01102)
Supplement: Supplementary file 3 [file Table_2.docx]

| Supplement 2 The other parameters related with live birth rate in different grade blastocysts | | | | | | | | |
| --- | --- | --- | --- | --- | --- | --- | --- | --- |
| Blastocyst quality grades | Goup1 (AA/AB/BA) | | Group2 (BB) | | Group3 (BC) | | Group4 (CB) | |
|  | Crude OR (95% CI) | *P* | Crude OR (95% CI) | *P* | Crude OR (95% CI) | *P* | Crude OR (95% CI) | *P* |
| Age(year) | 0.929(0.884-0.975) | 0.003 | 0.925(0.905-0.945) | <0.001 | 0.940(0.912-0.969) | <0.001 | 0.927(0.878-0.978) | 0.005 |
| Duration of infertility (years) | 0.973(0.903-1.048) | 0.973 | 0.934(0.905-0.964) | <0.001 | 0.986(0.941-1.032) | 0.540 | 0.976(0.913-1.044) | 0.483 |
| BMI (kg/m^2^) | 1.004(0.935-1.079) | 0.913 | 0.981(0.950-1.013) | 0.248 | 0.994(0.949-1.042) | 0.817 | 0.996(0.912-1.087) | 0.996 |
| Infertility type |  |  |  |  |  |  |  |  |
| Primary infertility | Ref |  | Ref |  | Ref |  | Ref |  |
| Secondary infertility | 0.868(0.578-1.302) | 0.493 | 0.828(0.681-1.007) | 0.058 | 0.787(0.591-1.048) | 0.101 | 0.998(0.602-1.652) | 0.992 |
| Infertility reason |  |  |  |  |  |  |  |  |
| Female | Ref |  | Ref |  | Ref |  | Ref |  |
| Male | 1.366(0.682-2.733) | 0.379 | 0.791(0.577-1.085) | 0.146 | 0.996(0.626-1.587) | 0.988 | 1.048(0.498-2.207) | 0.901 |
| Combined | 0.604(0.295-1.235) | 0.167 | 1.019(0.736-1.411) | 0.909 | 0.887(0.550-1.428） | 0.620 | 0.417(0.137-1.263) | 0.122 |
| Unknown | 1.486(0.831-2.655) | 0.181 | 0.974(0.743-1.276) | 0.849 | 0.803(0.538-1.198) | 0.282 | 1.037(0.549-1.960) | 0.910 |
| Number of 2PN (n) | 0.976(0.936-1.018) | 0.257 | 1.058(1.034-1.082) | <0.001 | 1.033(0.995-1.071) | 0.088 | 1.037(0.966-1.114) | 0.316 |
| Number of frozen blastocysts(n) | 0.940(0.808-1.093) | 0.419 | 1.184(1.050-1.334) | 0.006 | 1.056(0.823-1.354) | 0.670 | 1.251(0.667-2.346) | 0.486 |
| Insemination method |  |  |  |  |  |  |  |  |
| IVF | Ref |  | Ref |  | Ref |  | Ref |  |
| ICSI | 1.447(0.908-2.304) | 0.120 | 0.983(0.794-1.217) | 0.874 | 1.152(0.853-1.555) | 0.356 | 1.024(0.596-1.760) | 0.932 |
| Endometrial thickness (mm) | 1.125(1.027-1.232) | 0.011 | 1.043(1.000-1.087) | 0.048 | 1.039(0.976-1.106) | 0.235 | 0.892(0.793-1.003) | 0.056 |
| Endometrial preparation |  |  |  |  |  |  |  |  |
| Modified natural cycles | Ref |  | Ref |  | Ref |  | Ref |  |
| Hormone therapy cycles | 1.204(0.762-1.904) | 0.426 | 0.884(0.720-1.086) | 0.241 | 0.928(0.691-1.246) | 0.618 | 0.566(0.325-0.986) | 0.045 |
| Previous FET times |  |  |  |  |  |  |  |  |
| 0-1 | Ref |  | Ref |  | Ref |  | Ref |  |
| ≥2 | 0.691(0.418-1.141) | 0.148 | 0.869(0.686-1.102) | 0.247 | 1.173(0.833-1.651) | 0.360 | 0.915(0.486-1.724) | 0.784 |
| Treatment of year |  |  |  |  |  |  |  |  |
| 2010-2012 | Ref |  | Ref |  | Ref |  | Ref |  |
| 2013-2014 | 1.126(0.628-2.018) | 0.690 | 1.457(0.981-2.166) | 0.062 | 1.732(0.999-3.001) | 0.050 | 1.200(0.352-4.092) | 0.771 |
| 2015-2017 | 0.778(0.441-1.371) | 0.385 | 1.540(1.048-2.264) | 0.028 | 1.258(0.751-2.106) | 0.384 | 1.000(0.309-3.231) | >0.999 |
