# Supplementary material for: The Valuable Reference of Live Birth Rate in the Single Vitrified-Warmed BB/BC/CB Blastocyst Transfer: The Cleavage-Stage Embryo Quality and Embryo Development Speed
Source: Front Physiol. 2020 Sep 10;11:1102. doi: 10.3389/fphys.2020.01102 (PMC7511572; doi:10.3389/fphys.2020.01102)
Supplement: Supplementary file 4 [file Table_3.docx]

| Supplement 3 Crude and adjusted OR of clinical pregnancy rate in different grade blastocysts | | | | | |
| --- | --- | --- | --- | --- | --- |
|  |  | Crude OR (95% CI) | *P* | Adjusted OR (95%CI) | *P* |
|  |  | **Group1 (AA/AB/BA)** | | | |
|  | | | | | |
| Day3 assessment (cell number) | 6-7 (vs. < 6) | 1.239(0.753-2.038) | 0.400 | 1.299(0.762-2.215) | 0.336 |
|  | More than 7 (vs. <6) | 1.240(0.701-2.193) | 0.459 | 1.098(0.564-2.140) | 0.783 |
| Day3 assessment (embryo grade) | I-II (vs. III-IV) | 0.753(0.467-1.214) | 0.245 | 0.887(0.507-1.550) | 0.673 |
| Blastocyst frozen day | Day5 (vs. Day 6) | 1.131(0.750-1.706) | 0.556 | 1.218(0.740-2.004) | 0.438 |
| The degree of expansion | 3 (vs. 4) | 0.245(0.100-0.600) | 0.002 | 0.233(0.091-0.595) | **0.002** |
|  | 5 (vs. 4) | 0.614(0.270-1.394) | 0.243 | 0.674(0.276-1.643) | 0.385 |
|  | 6 (vs. 4) | 0.554(0.165-1.855) | 0.338 | 0.571(0.157-2.083) | 0.396 |
|  |  | **Group2 (BB)** | | | |
| Day3 assessment  (cell number) | 6-7 (vs. < 6) | 1.200(0.970-1.483) | 0.092 | 1.110(0.882-1.372) | 0.396 |
|  | More than 7 (vs. <6) | 1.119(0.862-1.452) | 0.400 | 0.976(0.736-1.295) | 0.868 |
| Day3 assessment (embryo grade) | I-II (vs. III-IV) | 0.970(0.731-1.287) | 0.831 | 0.931(0.686-1.263) | 0.644 |
| Blastocyst frozen day | Day5 (vs. Day 6) | 1.390(1.131-1.710) | 0.002 | 1.373(1.095-1.722) | **0.006** |
| The degree of expansion | 3 (vs. 4) | 0.327(0.132-0.811) | 0.016 | 0.350(0.135-0.906) | **0.030** |
|  | 5 (vs. 4) | 0.996(0.687-1.444) | 0.983 | 1.052(0.718-1.541) | 0.796 |
|  | 6 (vs. 4) | 1.199(0.675-2.128) | 0.535 | 1.427(0.804-2.534) | 0.224 |
|  |  | **Group3 (BC)** | | | |
| Day3 assessment (cell number) | 6-7 (vs. < 6) | 1.189(0.887-1.595) | 0.247 | 1.110(0.813-1.489) | 0.535 |
|  | More than 7 (vs. <6) | 1.248(0.840-1.855) | 0.273 | 1.175(0.781-1.770) | 0.439 |
| Day3 assessment (embryo grade) | I-II (vs. III-IV) | 0.633(0.378-1.062) | 0.084 | 0.702(0.401-1.229) | 0.215 |
| Blastocyst frozen day | Day5 (vs. Day 6) | 1.502(1.053-2.142) | 0.025 | 1.523(1.055-2.197) | **0.025** |
| The degree of expansion | 3 (vs. 4) | 0.683(0.309-1.509) | 0.346 | 0.918(0.381-2.210) | 0.848 |
|  | 5 (vs. 4) | 1.394(0.671-2.895) | 0.373 | 1.568(0.730-3.365) | 0.249 |
|  | 6 (vs. 4) | 3.186(0.580-17.496) | 0.182 | 3.620(0.706-18.556) | 0.123 |
|  |  | **Group4 (CB)** | | | |
|  |  |  |  |  |  |
| Day3 assessment (cell number) | 6-7 (vs. < 6) | 0.999(0.589-1.697) | 0.998 | 0.966(0.548-1.703) | 0.906 |
|  | More than 7 (vs. <6) | 1.661(0.885-3.117) | 0.114 | 1.470(0.745-2.899) | 0.266 |
| Day3 assessment (embryo grade) | I-II (vs. III-IV) | 0.828(0.330-2.078) | 0.687 | 0.977(0.326-2.932) | 0.967 |
| Blastocyst frozen day | Day5 (vs. Day 6) | 3.270(1.618-6.610) | 0.001 | 3.627(1.715-7.671) | **0.001** |
| The degree of expansion ^a^ | 5 (vs. 4) | 0.571(0.220-1.483) | 0.250 | 0.473(0.175-1.279) | 0.140 |
|  | 6 (vs. 4) | 0.285(0.063-1.301) | 0.105 | 0.284(0.064-1.266) | 0.099 |

Note: a: *P* value could not be calculated in expansion degree 3 subgroup because there are only 6 patients without clinical pregnancies (clinical pregnancy rate = 0/6). Crude OR=2.474E-13; Adjusted OR=4.712E-14. The bold P values of adjusted OR means statistical significance.
