# Supplementary material for: The Valuable Reference of Live Birth Rate in the Single Vitrified-Warmed BB/BC/CB Blastocyst Transfer: The Cleavage-Stage Embryo Quality and Embryo Development Speed
Source: Front Physiol. 2020 Sep 10;11:1102. doi: 10.3389/fphys.2020.01102 (PMC7511572; doi:10.3389/fphys.2020.01102)
Supplement: Supplementary file 5 [file Table_4.docx]

| Supplement 4 The other parameters related with clinical pregnancy rate in different grade blastocysts | | | | | | | | |
| --- | --- | --- | --- | --- | --- | --- | --- | --- |
| Blastocyst quality grades | Goup1 (AA/AB/BA) | | Group2 (BB) | | Group3 (BC) | | Group4 (CB) | |
|  | Crude OR (95% CI) | *P* | Crude OR (95% CI) | *P* | Crude OR (95% CI) | *P* | Crude OR (95% CI) | *P* |
| Age(year) | 0.978(0.932-1.025) | 0.353 | 0.939(0.920-0.959) | **<0.001** | 0.959(0.931-0.988) | **0.006** | 0.925(0.880-0.972) | **0.002** |
| Duration of infertility (years) | 1.019(0.942-1.102) | 0.636 | 0.943(0.915-0.971) | **<0.001** | 0.989(0.947-1.033) | 0.614 | 0.949(0.890-1.012) | **0.114** |
| BMI (kg/m^2^) | 1.033(0.957-1.114) | 0.405 | 0.984(0.953-1.015) | 0.313 | 1.010(0.966-1.056) | 0.662 | 1.013(0.936-1.096) | 0.754 |
| Infertility type |  |  |  |  |  |  |  |  |
| Primary infertility | Ref |  | Ref |  | Ref |  | Ref |  |
| Secondary infertility | 1.127(0.743-1.709) | 0.574 | 0.893(0.740-1.079) | 0.241 | 0.852(0.649-1.118) | 0.248 | 0.900(0.565-1.434) | 0.657 |
| Infertility reason |  |  |  |  |  |  |  |  |
| Female | Ref |  | Ref |  | Ref |  | Ref |  |
| Male | 1.312(0.643-2.675) | 0.455 | 0.803(0.595-1.085) | 0.153 | 1.142(0.734-1.774) | 0.556 | 1.056(0.529-2.105) | 0.878 |
| Combined | 0.887(0.446-1.763) | 0.733 | 0.927(0.674-1.274) | 0.641 | 0.995(0.636-1.555) | 0.981 | 0.445(0.172-1.155) | **0.096** |
| Unknown | 1.161(0.637-2.118) | 0.626 | 0.852(0.655-1.108) | 0.232 | 0.803(0.549-1.173) | 0.256 | 0.975(0.539-1.767) | 0.935 |
| Number of 2PN (n) | 0.966(0.925-1.009) | **0.122** | 1.042(1.019-1.065) | **<0.001** | 1.033(0.996-1.070) | **0.078** | 1.040(0.973-1.111) | 0.248 |
| Number of frozen blastocysts(n) | 0.972(0.823-1.148) | 0.742 | 1.151(1.025-1.293) | **0.017** | 1.008(0.791-1.283) | 0.950 | 1.343(0.738-2.444) | 0.334 |
| Insemination method |  |  |  |  |  |  |  |  |
| IVF | Ref |  | Ref |  | Ref |  | Ref |  |
| ICSI | 1.228(0.799-2.077) | 0.298 | 0.883(0.719-1.084) | 0.233 | 1.340(1.006-1.783) | **0.045** | 1.202(0.732-1.974) | 0.468 |
| Endometrial thickness (mm) | 1.117(1.016-1.228) | **0.022** | 1.039(0.997-1.083) | **0.070** | 1.041(0.980-1.106) | 0.189 | 0.927 (0.831-1.033) | 0.170 |
| Endometrial preparation |  |  |  |  |  |  |  |  |
| Modified natural cycles | Ref |  | Ref |  | Ref |  | Ref |  |
| Hormone therapy cycles | 1.449(0.906-2.318) | **0.112** | 0.906(0.743-1.105) | 0.328 | 0.944(0.714-1.247) | 0.684 | 0.766(0.469-1.253) | 0.289 |
| Previous FET times |  |  |  |  |  |  |  |  |
| 0-1 | Ref |  | Ref |  | Ref |  | Ref |  |
| ≥2 | 0.898(0.546-1.475) | 0.670 | 1.024(0.815-1.286) | 0.840 | 1.209(0.874-1.674) | 0.252 | 0.739(0.407-1.342) | 0.320 |
| Treatment of year |  |  |  |  |  |  |  |  |
| 2010-2012 | Ref |  | Ref |  | Ref |  | Ref |  |
| 2013-2014 | 1.415(0.795-2.520) | 0.238 | 1.419(0.980-2.056) | **0.064** | 1.926(1.140-3.254) | **0.014** | 1.222(0.388-3.850) | 0.732 |
| 2015-2017 | 0.858(0.492-1.497) | 0.590 | 1.305(0.911-1.868) | **0.147** | 1.423(0.872-2.320) | 0.158 | 1.146(0.384-3.424) | 0.807 |
